# Chronic Nasal Disease and Eustachian Tube Function: What Is the Role of Tubomanometry?

**DOI:** 10.3390/jcm13226731

**Published:** 2024-11-08

**Authors:** Sofia Anastasiadou, Polyzois Bountzis, Dimitrios-Evangelos Gkogkos, Petros Karkos, Jannis Constantinidis, Stefanos Triaridis, George Psillas

**Affiliations:** 1 Department of Medicine, Achepa University Hospital of Thessaloniki, Aristotle University of Thessaloniki, 541 24 Thessaloniki, Greece; pkarkos@aol.com (P.K.); janconst@otenet.gr (J.C.); triaridis@hotmail.com (S.T.); psill@otenet.gr (G.P.); 2 Department of Mathematics and Physics, Universita della Campania “Luigi Vanvitelli”, 81100 Caserta, Italy; pmpountzp@geo.auth.gr; 3 Department of Applied Informatics, University of Macedonia, 546 36 Thessaloniki, Greece; dgkogkos@uom.edu.gr

**Keywords:** Eustachian tube dysfunction, chronic nasal disease, tubomanometry

## Abstract

**Background/objectives**: Eustachian tube dysfunction (ETD) presents complex diagnostic challenges in otolaryngology, compounded by concurrent chronic nasal disease. Patient-reported outcome measures (PROMs) often assess ETD severity due to its elusive diagnosis. Tubomanometry (TMM) emerges as a promising diagnostic tool, yet its application alongside chronic nasal disease remains unclear. Our study aims to elucidate TMM’s role in ETD diagnosis within the context of chronic nasal diseases, integrating subjective assessments, clinical examination, and TMM results. **Methods**: A prospective observational study was conducted with patients suffering from ETD and chronic nasal disease allocated in three different groups according to their nasal pathology. Clinical examination, PROMs in the form of ETDQ-7, and NOSE questionnaires as well as TMM were performed. Results of the above subjective and objective measurements were analysed and correlated statistically to determine the value of TMM in chronic nasal disease patients. **Results**: All recruited patients suffered from ETD and chronic nasal disease, with similarly affected ETDQ-7 scores across all groups, while NOSE scores differed significantly based on the underground nasal pathology. TMM values confirm the presence of ETD in all three groups, confirming the role of TMM within this cohort. Interestingly, TMM values can still confirm the presence of ETD in patients with chronic nasal disease but cannot discriminate among chronic nasal pathology patients, making TMM a diagnostic tool with uniformity among the chronic nasal pathologies. **Conclusions**: ETD in individuals with chronic nasal disease presents distinct complexities, requiring a tailored diagnostic approach. In this context, a thorough clinical assessment, integrating ETDQ-7 and NOSE questionnaires, supplemented by TMM where accessible, is crucial to confirm diagnosis. This study confirms that TMM can diagnose ETD in all nasal pathology patients without being influenced by the nature of the disease. This research endeavours to refine diagnostic strategies, enriching clinical decision-making, and enhancing ETD management in patients suffering with chronic nasal diseases.

## 1. Introduction

Eustachian tube dysfunction (ETD) stands as a multifaceted and intricate challenge within the realm of otolaryngology, posing difficulties in both understanding its underlying mechanisms and establishing precise diagnostic criteria [1]. Concurrent chronic nasal disease complicates the clinical picture as it adds more factors that contribute to similar symptoms. Chronic nasal disease significantly affects the nasopharynx as well as the ET orifices and plays a significant role in ETD presentation and symptoms. Patient reported outcome measures (PROMs) are usually used to assess the severity of ETD. Tubomanometry (TMM) is a relatively novel objective diagnostic method of ETD and is the diagnostic tool of choice where it is available [2,3,4]. But what happens with TMM when there is concurrent chronic nasal disease remains vaguely unexplored. Similarly, which pathology to correct first, either the nasal disease or the ETD itself or even perform synchronous procedures, has not yet been discussed adequately in the literature. Our study aims to contribute valuable insights into the role of TMM in the diagnostic algorithm for ETD within the context of chronic nasal diseases. Integration of subjective symptom assessments, clinical examinations, and TMM results promises a comprehensive understanding of Eustachian tube function in this complex patient population.

Chronic nasal disease is multifaceted and can be expressed as chronic rhinitis, nasal septal deviation (NSD), as well as chronic rhinosinusitis (CRS). Based on the area of the nose affected, there are multiple different nasal pathologies, functional, structural, or inflammatory. In many cases, more than two pathologies co-exist with characteristics of both anatomical and inflammatory changes that contribute to the chronic nasal disease. It is occasionally challenging to discriminate between the two, and this represents one of the nuances in determining the relation between ETD and chronic nasal disease. This study aims to determine the reliability of TMM in patients with chronic nasal diseases and to identify if specific nasal pathologies, such as chronic rhinitis, NSD, or CRS with polyps, have a greater impact on TMM findings and thus on the diagnosis of ETD. Additionally, it seeks to correlate PROMs, specifically the Eustachian Tube Dysfunction Questionnaire-7 (ETDQ-7) and NOSE questionnaire scores [5], with TMM results to compare subjective and objective assessments of ETD in this particular patient population.

## 2. Methods

In this study, ear, nose, and throat (ENT) patients were recruited from outpatient or pre-operative assessment units that suffered with ETD and concurrent nasal pathology in Achepa University Hospital of Thessaloniki. They were provided with information leaflets, and the researchers had individual meetings with each patient to explain the process of the study. A consent form was signed by all patients after adequate discussion and information was provided. The data collection lasted 15 months and was also approved by the Committee of Bioethics and Deontology of the Hospital. The patients were allocated in three different groups according to their chronic nasal pathology. The first group presented with chronic rhinitis, the second one had NSD, and the third one chronic rhinosinusitis (CRS) with polyps. Patients in the septal deviation group were also subgrouped, taking into consideration the Baumann et al. six-type classification of septal deviation [6,7]. In the group with CRS with polyps, there were multiple polyps instead of one single polyp (e.g., antrochoanal polyp) and polyp location in the nose was documented as well. Each patient in every group had only one of these characteristic findings on examination, and there was no overlap of the chronic nasal pathologies.

The sample size was calculated based on the total population of chronic nasal disease patients that were reviewed in the outpatient department within a year, with a confidence interval of 90%, a standard deviation of 0.5%, and a margin of error within 5–7%. This is consistent with the current literature findings and ensures reliability of the results as well as represents a realistic sample size based on the overall population size in the outpatient department.

**Figure 1:**
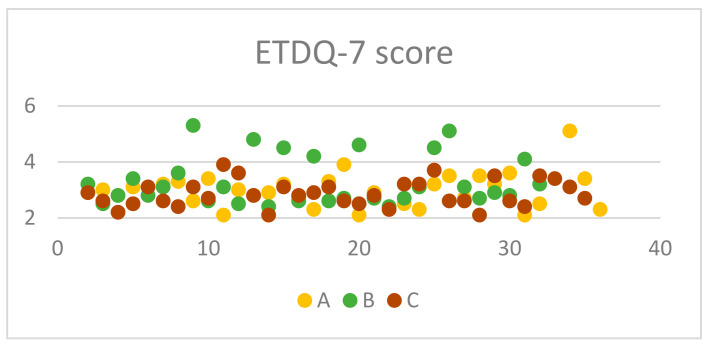
ETDQ7 score among groups.

**Figure 2:**
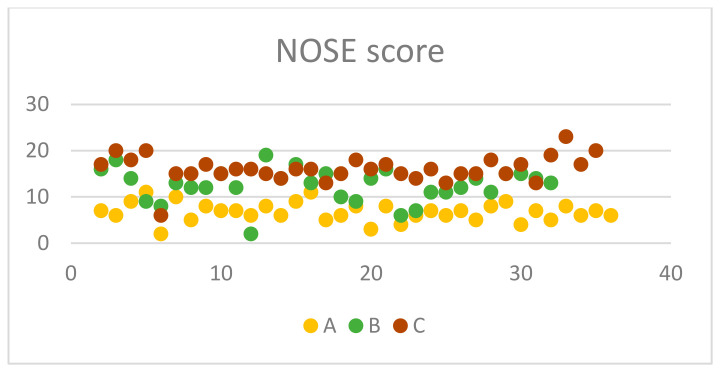
NOSE score among groups.

The presence of ETD and chronic nasal disease was established using both subjective and objective measurement tools. Patient reported outcome measures were utilised to recruit patients to the first phase of the trial. This involved a comprehensive evaluation employing the ETDQ-7 as well as the NOSE questionnaire [5]. A minimum score of 2.1 for ETDQ-7 and 4 for NOSE was required for a patient to be considered eligible for the study. It appears that the majority of the participants lie between 2.5 and 4.0 for ETDQ-7 and 5 and 20 for NOSE accordingly (Figure 1 and Figure 2). According to the literature, this is the minimum score for each questionnaire to indicate pathology [5,8]. Inclusion criteria apart from the PROMs scores were the following: specific nasal pathology to be eligible for one of the three groups, examination findings of ETD, either retracted tympanic membrane or Type C tympanogram, age > 18 years, ETD symptoms for more than 12 weeks and for Group C only, middle meatal polyps [9]. Exclusion criteria were previous ENT operations; severe ear pathology such as ear drum perforation, tympanosclerosis, or cholesteatoma; adenoid hypertrophy; postnasal space lesions such as nasopharyngeal tumours; previous head and neck radiotherapy; congenital abnormalities; temporomandibular joint dysfunction; and various polyp locations for Group C. Temporomadibular joint dysfunction significantly mimics ETD symptoms, and patients in Group C had only polyps occupying their middle meatus. Inclusion and exclusion criteria as well as demographics can be found in Table 1 and Table 2.

Objective examination findings were utilised including otoscopy, anterior rhinoscopy, and flexible nasoendoscopy. These were used to determine whether they fulfil the inclusion criteria as well as which chronic nasal condition they suffer from. Tympanometry was also used as an adjunct to diagnose ETD. Either tympanometry or otoscopic findings of tympanic membrane retraction confirmed the ETDQ-7 score in order to progress to recruitment of the patients to the study.

**Figure 3:**
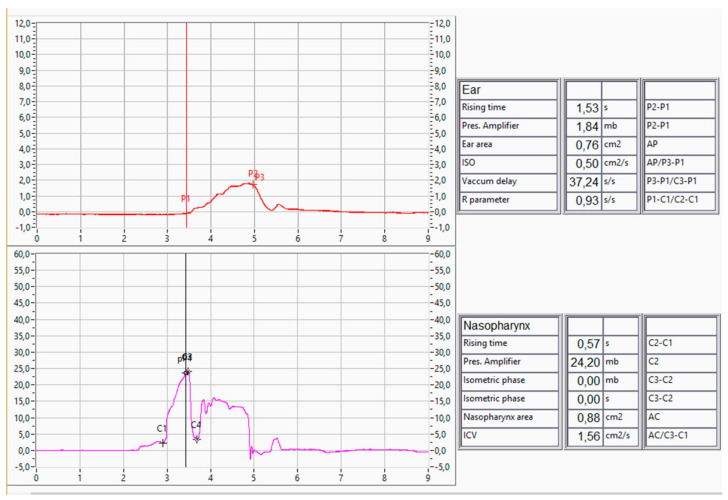
Normal TMM curves with measurements, right ear.

**Figure 4:**
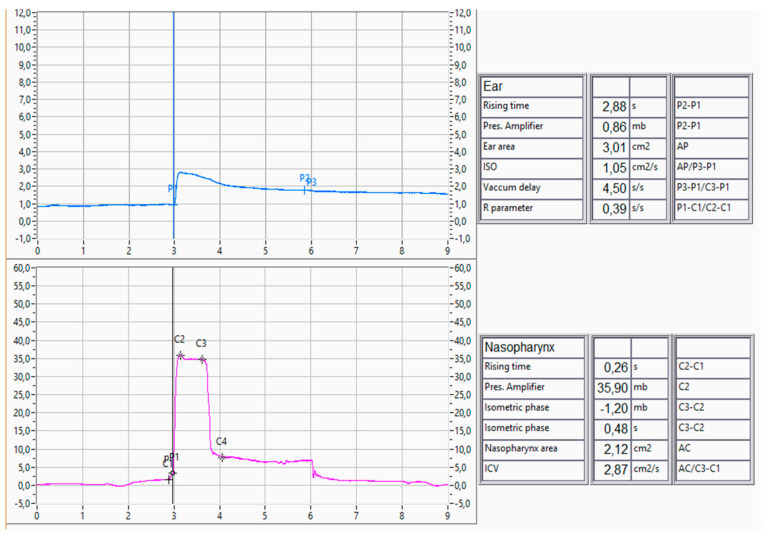
Normal TMM curves with measurements, left ear.

Patients were divided in three groups, with 35 patients in the chronic rhinitis group (Group A), 31 patients in the NSD group (Group B), and 34 patients in the CRS group (Group C). Recruitment was stopped as an appropriate number for each group was reached, according to similar studies in the literature [10,11]. The patients allocated to Group A firstly presented with pathological NOSE scores that suggested chronic nasal obstruction. Subsequently, the patients underwent clinical examination to identify the cause of the pathological NOSE score. Chronic rhinitis was defined as nasal mucosa irritation mostly attributed to allergic rhinitis. The nose appeared congested with evidence of clear discharge and erythematous mucosa. The patients did not have any other significant pathology, no septal deviation, or nasal polyps. Group C patients were diagnosed with CRS with nasal polyps that were present in the middle meatus without causing complete nasal obstruction or fully occupying the inferior meatus. Patients underwent tubomanometry (TMM) to assess the severity of their ETD status. The R-value, a parameter derived from TMM, provides meaningful insights into ET function. An R-value ≤ 1 signifies a regular and timely opening of the ET, while an R-value > 1 indicates a delayed opening. The absence of a definable R-value suggests no detectable opening of the ET. Normal TMM findings are represented in Figure 3 and Figure 4. This quantitative measure enhances the diagnostic precision in identifying chronic obstructive ET dysfunction. Finally, TMM scores were correlated to their specified nasal pathology as well as their PROMS, ETDQ-7, and NOSE scores. 

Statistical analysis was carried out using SPSS Statistics version XX software (IBM Corp., New York, NY, USA). The Spearman rank correlation coefficient was used, which is a non-parametric measure, to assess the strength and direction of the relationship between NOSE and ETDQ-7 scores with R, C2, and C2-C1. Values closer to 1 indicate strong positive correlation between the compared values, whereas values closer to -1 indicate strong negative correlation, respectively. We further performed the two-sided Spearman rank correlation test to explore statistically significant interactions among the variables. The comparisons among the groups were statistically analysed using the Kruskal–Wallis test. Dunn’s post-hoc test was used to conduct pairwise comparisons between groups after finding a significant result from the Kruskal–Wallis test. A *p*-value < 0.05 was considered to indicate statistical significance.

## 3. Results

**Figure 5:**
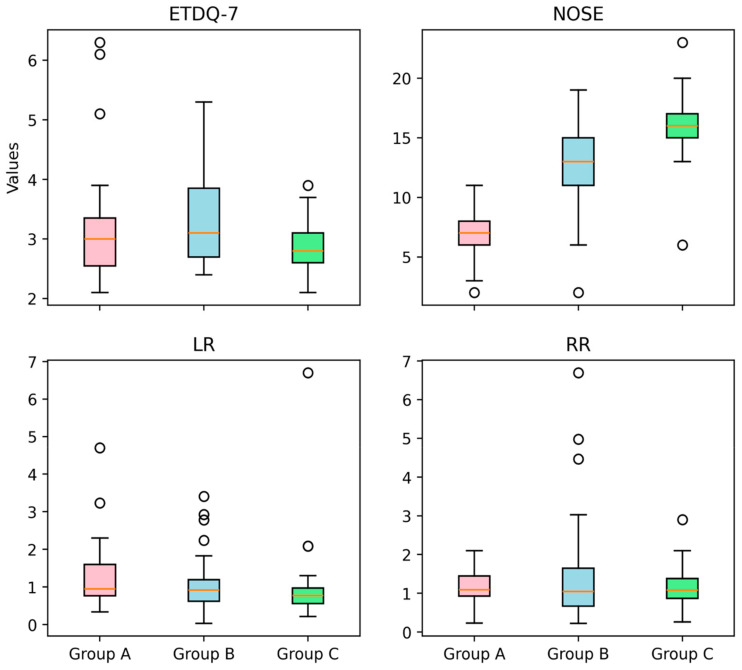
ETDQ-7, NOSE, LR, and RR scores among groups.

**Figure 6:**
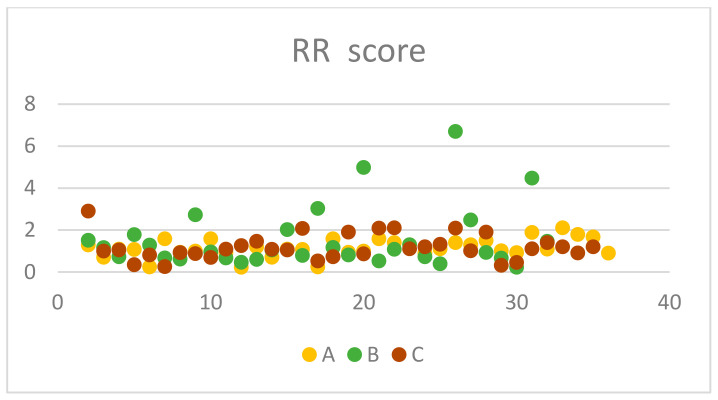
RR score among groups.

**Figure 7:**
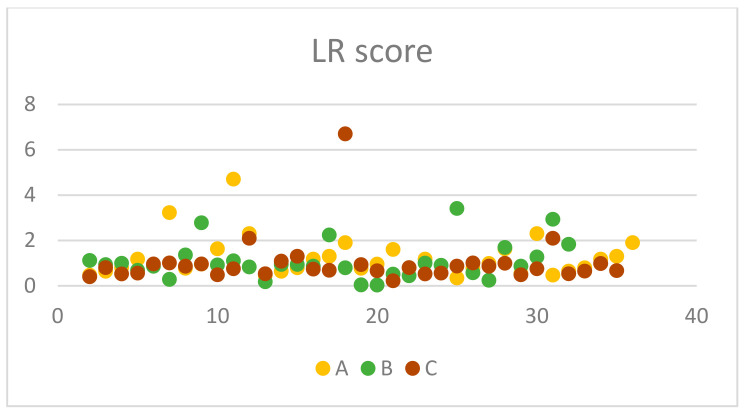
LR score among groups.

From the 35 chronic rhinitis patients enrolled in Group A, 14 (40%) were males and 21 (60%) females. The mean (standard deviation) age of the enrolled patients was 41.2 (14.8) years old (Table 2). Clinical measures with otoscopy and rhinoscopy showed pathological outcomes with rhinetic swollen nasal mucosa and hypertrophic inferior turbinates with tympanic membrane retraction or tympanometry type C. The ETDQ-7 and NOSE scores were 3.14 and 6.77, respectively, which are both within the abnormal values (Table 3). TMM was therefore performed to assess Eustachian tube (ET) function bilaterally, with corresponding right and left R-scores equal to 1.15 (0.43) and 2.42 (4.49). Both these R scores indicate ETD with the ET to remain closed most of the time. TMM data are represented in Table 4 and are summarised in Figure 5, Figure 6 and Figure 7. This confirms that TMM can correctly diagnose ETD in chronic rhinitis patients and also that TMM values correlate to clinical symptoms and PROMs.

Similarly, in Group B, 31 patients with NSD were recruited, 18 (58.1%) were male and 13 (41.9) were females. The mean age of the group was 36 with a standard deviation of 9.09 (Table 2). Clinical examination with otoscopy and rhinoscopy showed NSD with associated tympanic membrane retraction or tympanometry type C. The ETDQ-7 score was 3.31 and NOSE score was 12.48, both indicating severe ET and nasal symptoms (Table 3). From a total of 31 patients, 11 patients had Type II as per Baumann et al. [6] NSD and 20 patients had type III. TMM values were right-sided R 1.54 and left-sided R score 1.08. The results also confirm that the side of the NSD corresponds to the most pathological R score as the contralateral side shows a milder ETD with a lower R score closer to 1. This also reflects to what the patient reported as the most heavily congested side of the nose. TMM data are represented in Table 5 and are summarised in Figure 5, Figure 6 and Figure 7. Therefore, TMM can be reliably used in NSD patients and can also be correlated with the side of the obstruction caused by the existing NSD.

Finally, in Group C, 34 patients with CRS with polyps were recruited with a 41.1% male and 55.9% female ratio. The mean age of the patients was 48 with a standard deviation of 12.3 (Table 2). Clinical examination once again revealed ETD otoscopically, or tympanometry confirmed and flexible nasoendoscopy revealed nasal polyposis with polyps occurring in the middle meatus and no significant NSD. The ETDQ-7 score was 2.85 and NOSE score was 16.03, both indicating significant nasal congestion and ETD (Table 3). TMM data are represented in Table 6 and are summarised in Figure 5, Figure 6 and Figure 7. The right-sided R score in TMM was 1.18, while the left-sided R score was 0.99. This again indicates ETD; however, the right side appears closer to normal values compared to the left.

**Figure 8:**
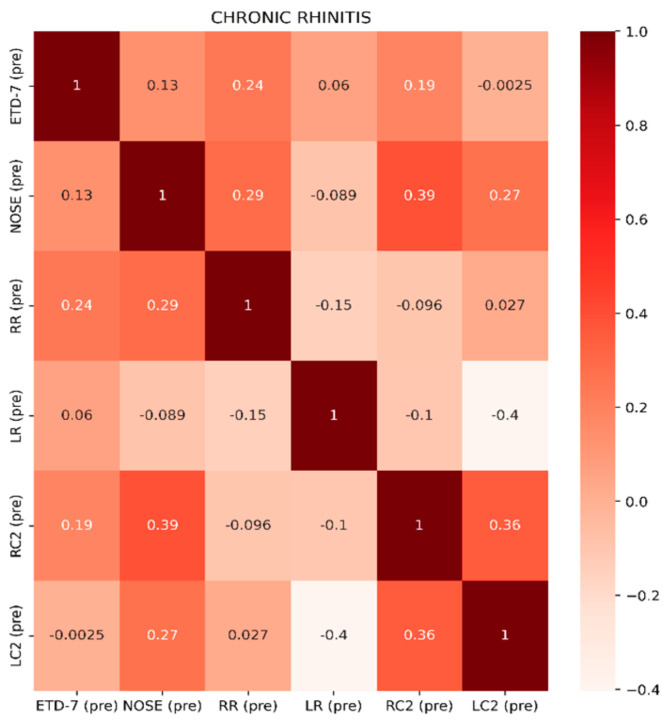
Spearman correlation coefficients among features ETD-7, NOSE, RR, LR, RC2, and LC2 for Group A.

**Figure 9:**
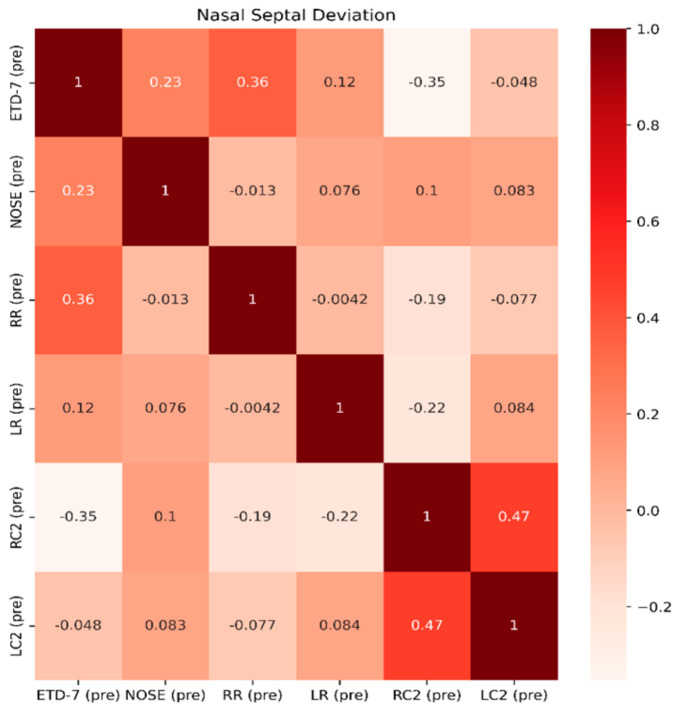
Spearman correlation coefficients among features ETD-7, NOSE, RR, LR, RC2, and LC2 for Group B.

**Figure 10:**
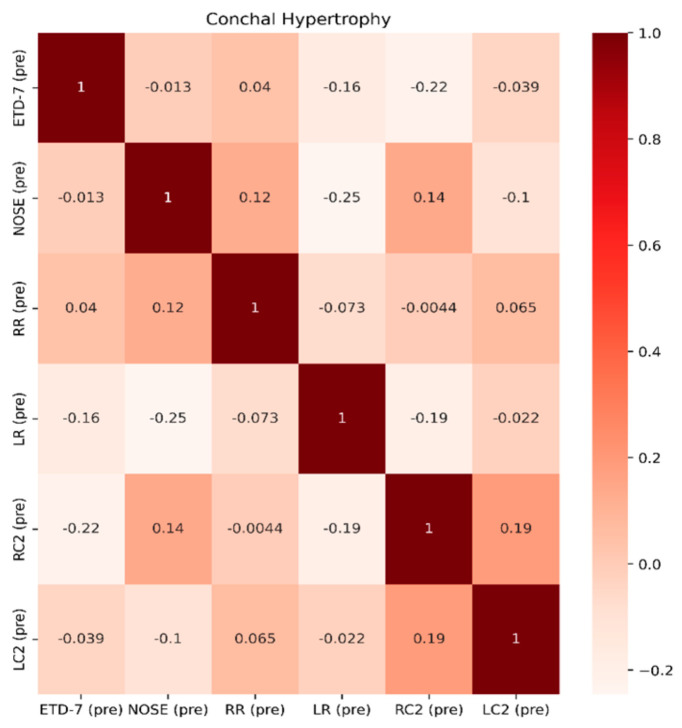
Spearman correlation coefficients among features ETD-7, NOSE, RR, LR, RC2, and LC2 for Group C.

In terms of the PROMs used in the study, it was considered significant to correlate these to the clinical findings as well as the TMM values. Also, it was interesting to identify which nasal pathology affects more TMM values, such as R, C2-C1, and C2 values that might indicate significant ETD. PROM results among groups are presented in Figure 6, Figure 7 and Figure 8. In Group A, it is evident that there is a statistically significant positive correlation between NOSE scores and R values (rho = 0.39, *p* = 0.021). This means that patients suffering from chronic rhinitis and higher NOSE scores come in accordance with higher R as well as increased pressure in the nasopharynx (C2) (Figure 8). In group B, ETDQ-7 and RR features show a stronger correlation compared to NOSE and TMM values (rho=0.36, *p* =0.054). This reflects that ETDQ-7 is more reliable in this cohort of patients to be correlated with TMM results compared to the NOSE questionnaire. NSD significantly affects the structure of the nose, and NOSE scores are higher the worse the deviation is (Figure 9). Last but not least, in Group C, the correlations of the tests are even less significant, showing that TMM confirms the presence of ETD, but TMM values are not strongly correlated with PROMs values (Figure 10). Overall PROMs scores are illustrated in Figure 7 and Figure 8.

To compare the two questionnaire’s scores among the three groups, the analysis was conducted via the independent-samples Kruskal–Wallis test and aimed to evaluate the distribution of ETD-7 and NOSE scores across the three groups. It appears that the *p* value was calculated to be 0.807 for ETDQ-7, indicating uniformity in ETD among patients in the three groups. In contrast, the *p* value for NOSE was calculated to be 0.001, which indicates that NOSE questionnaire scores were significantly different among patients in the three different groups. After performing the independent-samples Kruskal–Wallis test among the NOSE results of each group, it was evident that NOSE was affected differently. Performing the post-hoc Dunn test showed that the most outstanding NOSE questionnaire scores belong to Group A, as these patients have milder symptoms compared to Group B and C (Table 7a,b). This is reflected in the mean NOSE questionnaire values that are 6.77, 12.48, and 16.03, respectively, for Groups A, B, and C (Table 3). Subsequently, this study confirmed the value of ETDQ-7 and NOSE questionnaires in the diagnostic pathway of ETD.

The independent-samples Kruskal–Wallis test which is a non parametric test comparing independent figures among groups, was also performed to assess the relation of TMM values, R, C2, and C2-C1 to identify possible discrepancies among the three groups. It appears that there is no statistically significant difference among the three groups in terms of TMM measurements. All patients with ETD appear to have coherent R, C2-C1, and C2 values, which might imply that nasal pathology can cause ETD irrespectively of its nature. In detail, the nasal pathology significantly affects the ET function and specifically how high the pressure can build up in the nasopharynx and how quickly this pressure rises in the area. However, there is no statistically significant difference among the three groups and their TMM values, indicating that TMM cannot discriminate among different chronic nasal pathology.

## 4. Discussion

The critical evaluation of TMM gains heightened significance when ETD coexists with chronic nasal diseases, including chronic rhinitis, NSD, or CRS with polyps [10,11,12,13]. The interplay between these conditions introduces additional layers of complexity and considerations that must be carefully weighed when utilising TMM in the diagnostic process.

Chronic nasal disease, characterised by persistent inflammation and anatomical variations, can exert a profound influence on Eustachian tube function. In the context of allergic rhinitis, the mucosal inflammation and increased nasal secretions may impact the patency of the Eustachian tube, contributing to the manifestation of ETD symptoms. Similarly, NSD can alter the aerodynamics of the nasal passages, influencing the pressure dynamics within the Eustachian tube [10,13]. Chronic rhinosinusitis with polyps further complicates the picture by introducing inflammatory changes and potential obstruction within the nasal and sinus cavities [11]. To our knowledge, there is no study in the current literature that correlates different nasal pathologies and compares how these affect PROMs (ETDQ-7, NOSE questionnaires) with objective TMM measurements.

### 4.1. Patient-Reported Outcome Measures—PROMS

Patient-reported outcome measures (PROMs) offer a valuable window into patient experiences, particularly when objective tests are lacking [14,15]. These questionnaire-based tools capture subjective symptoms and quality-of-life impacts, providing quantitative data that complement traditional clinical history-taking. In the context of Eustachian tube dysfunction (ETD), where a gold-standard objective test remains elusive, one specific PROM has emerged: the seven-item ETD Questionnaire (ETDQ-7) by McCoul et al. [5]. However, limitations exist. While the ETDQ-7 has gained traction in clinical and research settings, it struggles to distinguish between obstructive and patulous ETD subtypes. Additionally, its specificity drops significantly when tested against a broader population with mixed otolaryngological conditions [9,16]. This suggests that initial assessments using healthy control groups or those with unrelated pathologies may have overestimated its diagnostic accuracy. Although, while not ideal for diagnosis, this tool has been widely utilised to determine the presence and quantify the symptoms of ETD but also to monitor symptom changes and treatment response in patients with diagnosed ETD [17]. In this study, it appears that ETDQ-7 confirms the presence of ETD alongside clinical findings with otoscopy and tympanometry. It is also evident that ETDQ-7 reflects the severity of ETD, as higher scores of ETDQ-7 are correlated with pathological TMM measurements, with higher R, C2, and C2-C1 values. However, ETDQ-7 is not capable to discriminate among different nasal pathologies, as it appears to be equally affected in all three groups.

Another PROM was used in this study to identify nasal pathology and discriminate patients with severe nasal symptoms. The NOSE questionnaire was developed by Stewart et al. [18] and is used vastly in research as well as clinical practice in the field of rhinology. It can effectively quantify nasal symptoms and act as an adjunct to assess severity of the nasal condition. It has been validated in the paediatric population as well and has recently been revalidated in large groups of the population [19,20]. In this study, it was used to assess the severity of nasal symptoms, and in all three groups of patients, it showed a pathological score. After statistical analysis, it appears that NOSE scores are correlated with TMM values with higher NOSE scores to pair with abnormal R, C2, and C2-C1 scores. Even though the NOSE questionnaire is not directly linked to ETD, there is a clear relation between the two, taking into consideration the TMM findings. It is also evident that NOSE scores are different across the three groups, with the lower scores represented in chronic rhinitis patients and statistically different to higher ones that belong to chronic rhinosinusitis patients.

### 4.2. Tubomanometry—TMM

Tubomanometry (TMM), as an objective diagnostic tool, must be scrutinised for its applicability in the presence of these coexisting conditions. There has already been significant discussion around the reliability and applicability of TMM to investigate ETD in an otherwise normal population [21]. The reliability and interpretability of TMM results may be affected by the altered nasal anatomy, dynamic nasal airflow patterns, and variable degrees of mucosal inflammation [10,11,12]. Moreover, patient discomfort and compliance during TMM may be exacerbated in individuals with chronic nasal diseases. The presence of nasal congestion, rhinorrhea, or sinonasal discomfort can influence a patient’s ability to execute the required manoeuvres and may introduce confounding variables into the assessment. In this study, three different measurements were performed for every patient, in 30, 40, and 50 mbar as indicated in the literature, and the mean of all values was used for statistical analysis [2,22]. TMM was performed on all patients across all three nasal pathology groups, and the values were correlated with their ETD as well as nasal pathology. As previously mentioned, the interest of the study is focused on the R parameter, C2, and C2-C1. An R-value ≤ 1 signifies a regular and timely opening of the ET, while an R-value > 1 indicates a delayed opening. The absence of a definable R-value suggests no detectable opening of the ET. The value C2 indicates the highest-pressure measurement in the nasopharynx, and the C2-C1 indicates the speed with which the pressure rises in the nasopharynx during the measurements. A normal TMM measurement is shown in Figure 3 and Figure 4. In this study, TMM values show statistically significant correlation with ETDQ-7 and NOSE measurements, confirming the presence of ETDQ-7 in all groups. The study also implies that TMM values are not affected in the specific population of chronic nasal disease, and it can still reflect the ETD pathology with reliably abnormal, R, C2, and C2-C1 values. This has not yet been discussed in the literature, and it is important to conduct larger studies with multiple chronic nasal pathology patients to verify our results.

### 4.3. Which Nasal Disease Affects the ET More?

This study aimed to shed light on the relationship between chronic nasal disease and ETD. Patients with chronic rhinitis which presents as mostly allergic have less pathological NOSE scores compared to those with NSD, and the latter group has better NOSE scores compared to the CRS with polyps. ETDQ-7 scores that reflect the severity of ETD symptoms appear similar to all three groups, implying that the nasal condition does not subjectively affect what the patients with ETD feel. TMM measurements also show high correlation with subjective measurement outcomes and also appear to be consistently pathological across all groups of patients. TMM can be used to confirm the presence of ETD in chronic nasal disease patients and is not altered based on the nature of the chronic nasal pathology. This is also confirmed in the literature; however, studies conducted to date discuss a single nasal pathology and especially nasal septal deviation and chronic nasal polyposis. Our study aims to compare multiple cohorts of patients and confirms the valid use of TMM among these groups.

However, this study shows that TMM cannot discriminate among different nasal pathologies. This is interpreted as either that chronic nasal disease influences the function of ET irrespectively of the nature of the pathology as long as the symptoms are obstructive, or that possibly TMM is not the appropriate investigation to discriminate among different nasal pathologies. There is no other study in the literature to compare our results, so TMM can reliably be used for all chronic nasal disease patients; however, TMM values do not reflect each nasal pathology. Other rhinomanometric tests might be recommended as adjuncts to correlate with ETD and its severity in various nasal pathologies.

### 4.4. Limitations

This study has specific limitations that need to be mentioned and presented. First, the sample size is limited, and this might affect the generalisation of the results. Second, overlapping of symptoms as well as nuances of TMM measurements and difficulties while performing the technique might have also influenced the results unintentionally. This might be the explanation for two separate extreme values of TMM, especially in Group C, that were considered inaccurate and were not included in the statistical analysis. Finally, the results should be interpreted with care and should inspire further bigger trials to be able to draw more reliable conclusions regarding chronic nasal disease and TMM measurements.

## 5. Conclusions

ETD in patients with chronic nasal disease are of particular interest, as these individuals often face unique challenges in ET function. In light of these considerations, a comprehensive diagnostic approach to ETD in the context of chronic nasal diseases should encompass a thorough clinical evaluation, incorporating a combination of subjective symptoms with ETDQ-7 and NOSE questionnaires. This study shows that TMM can be reliably used in this cohort of patients to diagnose ETD irrespective of their nasal pathology. Through this research, diagnostic approaches are refined to enhance clinical decision-making, and ultimately improve the management of ETD in individuals suffering with chronic nasal diseases.

## Figures and Tables

**Table 1:** Inclusion and exclusion criteria.

Inclusion Criteria	Exclusion Criteria
Age > 18 years old	Previous ENT surgery
ETD symptoms > 12 weeks ETDQ-7 > 2.1	Severe ear pathology (cholesteatoma, etc.)
No ENT surgery previously	Adenoid hypertrophy
No recent medication applied Retracted tympanic membrane and/or Type C tympanogram	Postnasal space lesion
Chronic nasal disease	Previous H&N area radiotherapy

**Table 2:** Demographic characteristics among patient groups.

		Group A	Group B	Group C
Gender	M	40% (n=14)	58.1% (n=18)	41.1% (n=15)
	F	60% (n=21)	41.9% (n=13)	55.9% (n=19)
Age	Mean (SD)	41.2 (14.8)	36 (9.09)	48 (12.3)

**Table 3:** PROMs and TMM values among groups.

	Group A	Group B	Group C
	Mean (SD)		
ETDQ-7	3.14 (0.96)	3.30 (0.86)	2.85 (0.46)
NOSE	6.77 (2.03)	12.48 (3.63)	16.02 (2.84)
LR	2.42 (4.94)	1.08 (7.92)	0.99 (1.08)
RR	1.15 (0.43)	1.54 (10.28)	1.18 (0.59)
LC2	28.74 (5.48)	26.41 (7.92)	27.23 (4.23)
RC2	26.25 (7.43)	22.87 (10.28)	22.94 (9.22)

**Table 4:** Statistics of tubomanometry values for Group A.

	ETD-7	NOSE	RR	RC2	LR	LC2
Std. Deviation	1.0490	2.295	0.418261738030	7.2381	3.60317940615	5.7265
Variance	1.100	5.268	0.175	52.390	12.983	32.793
Range	5.1	10	1.990	24.9	21.869	26.3
Minimum	1.2	1	0.1100	11.0	0.030	10.5
Maximum	6.3	11	2.10	35.9	21.900	36.8

**Table 5:** Statistics of tubomanometry values for Group B.

	ETD-7	NOSE	RR	RC2	LR	LC2
Std. Deviation	1.0282	5.135	1.167849634311	9.9750	1.01687805989	7.7359
Variance	1.057	26.372	1.364	99.501	1.034	59.845
Range	4.2	18	6.520	39.1	6.670	33.3
Minimum	1.1	1	0.180	0.6	0.030	1.4
Maximum	5.3	19	6.700	39.7	6.70	34.7

**Table 6:** Statistics of tubomanometry values for Group C.

	ETD-7	NOSE	RR	RC2	LR	LC2
Std. Deviation	0.4613	2.844	0.59574	9.2261	1.08088	4.2390
Variance	0.213	8.090	0.355	85.121	1.168	17.969
Range	1.8	17	2.64	36.1	6.48	18.9
Minimum	2.1	6	0.26	0.3	0.22	16.6
Maximum	3.9	23	2.90	36.4	6.70	35.5

**Table 7:** (**a**) The *p*-values of the Dunn post-hoc analysis with Bonferroni adjustment. (**b**) Independent-samples Kruskal–Wallis test for questionnaires.

(a)			
Group	Chronic Rhinitis	NSD	CRS
A	1	1.95 × 10^−5^	1.78 × 10^−15^
B		1	0.003
C			1
**(b)**			
**Features**		***p*** **-Value**	
ETD-7~Groups		0.191	
NOSE-Groups		0.000	

## Data Availability

All new data generated in this study are available upon request.
